# Fluoxetine Induced Suicidal Erythrocyte Death

**DOI:** 10.3390/toxins5071230

**Published:** 2013-07-15

**Authors:** Kashif Jilani, Sigrid Enkel, Rosi Bissinger, Ahmad Almilaji, Majed Abed, Florian Lang

**Affiliations:** 1 Department of Physiology, University of Tuebingen, Gmelinstr. 5, Tuebingen 72076, Germany; E-Mails: kashif_cbc@yahoo.com (K.J.); ahmad_almilaji@yahoo.co.uk (A.A.); dr.magd81@hotmail.com (M.A.); 2 Zentrum für Klinische Transfusionsmedizin, Otfried-Müller-Straße 4/1, Tuebingen 72076, Germany; E-Mail: sigrid.enkel@med.uni-tuebingen.de

**Keywords:** phosphatidylserine, fluoxetine, calcium, cell volume, eryptosis

## Abstract

The antidepressant fluoxetine inhibits ceramide producing acid sphingomyelinase. Ceramide is in turn known to trigger eryptosis the suicidal death of erythrocytes characterized by cell shrinkage and exposure of phosphatidylserine at the erythrocyte surface. Ceramide is effective through sensitizing the erythrocytes to the pro-eryptotic effect of increased cytosolic Ca^2+^ activity ([Ca^2+^]_i_). In nucleated cells, fluoxetine could either inhibit or stimulate suicidal death or apoptosis. The present study tested whether fluoxetine influences eryptosis. To this end cell volume was estimated from forward scatter, phosphatidylserine exposure from annexin V binding, hemolysis from hemoglobin release and [Ca^2+^]_i_ from Fluo-3 fluorescence intensity. As a result, a 48 h exposure of erythrocytes to fluoxetine (≥25 µM) significantly decreased forward scatter, increased annexin V binding and enhanced [Ca^2+^]_i_. The effect on annexin V binding was significantly blunted, but not abolished, in the absence of extracellular Ca^2+^. In conclusion, fluoxetine stimulates eryptosis, an effect at least in part due to increase of cytosolic Ca^2+^ activity.

## 1. Introduction

Fluoxetine is a widely used antidepressant [1,2], which has been shown to inhibit acid sphingomyelinase [3,4]. Sphingomyelinase activity generates ceramide, which in turn triggers suicidal death of nucleated cells [5] and erythrocytes [6]. In view of the inhibitory effect of fluoxetine on the sphingomyelinase, fluoxetine could be expected to inhibit suicidal death of erythrocytes or eryptosis [6]. As a matter of fact, fluoxetine has been shown to inhibit suicidal death or apoptosis of nucleated cells [7,8,9]. On the other hand, fluoxetine has been shown to trigger or foster apoptosis of nucleated cells [10,11]. The paper thus explored whether fluoxetine influences eryptosis.

Eryptosis is characterized by erythrocyte shrinkage and translocation of phosphatidylserine to the erythrocyte cell membrane surface [12]. Ceramide triggers eryptosis by sensitizing erythrocytes to the effects of increased cytosolic Ca^2+^ activity ([Ca^2+^]_i_) [6]. [Ca^2+^]_i_ may increase due to Ca^2+^ entry through Ca^2+^ permeable cation channels [13,14]. The increase of [Ca^2+^]_i _is followed by cell shrinkage due to activation of Ca^2+^ sensitive K^+^ channels [15], cellular K^+^ exit, hyperpolarization of the cell membrane, Cl^−^ exit and thus cellular loss of KCl with osmotically obliged water [16]. Increased [Ca^2+^]_i _further triggers translocation of phosphatidylserine to the erythrocyte surface thus disrupting the phosphatidylserine asymmetry of the erythrocyte cell membrane [17]. Eryptosis is further elicited by energy depletion [18] and caspase activation [19,20,21,22,23]. Signaling governing eryptosis or erythrocyte survival includes several kinases, such as AMP activated kinase AMPK [14], cGMP dependent protein kinase [24], Janus activated kinase JAK3 [25], casein kinase [26,27], p38 kinase [28], PAK2 kinase [29] as well as sorafenib [30] and sunifinib [31] sensitive kinases.

The present study explored whether fluoxetine impacts on eryptosis. To this end, [Ca^2+^]_i_, cell volume and phosphatidylserine exposure at the cell surface were determined prior to and following fluoxetine exposure. As a result, fluoxetine increases [Ca^2+^]_i_, decreases erythrocyte volume and enhances the phosphatidylserine abundance at the erythrocyte surface. 

## 2. Methods

### 2.1. Erythrocytes, Solutions and Chemicals

Leukocyte depleted erythrocytes were kindly provided by the blood bank of the University of Tübingen. The study is approved by the ethics committee of the University of Tübingen (184/2003V). Erythrocytes were incubated *in vitro* at a hematocrit of 0.4% in Ringer solution containing (in mM) 125 NaCl, 5 KCl, 1 MgSO_4_, 4-(2-hydroxyethyl)-1-piperazineethanesulfonic acid (HEPES), 5 glucose, 1 CaCl_2_; pH 7.4 at 37 °C for 48 h. Where indicated, erythrocytes were exposed to fluoxetine (Enzo, Lörrach, Germany) at the indicated concentrations. In Ca^2+^ free Ringer solution, 1 mM CaCl_2_ was substituted by 1 mM glycol bis(2 aminoethylether)-*N*,*N*,*N*',*N*'-tetraacetic acid (EGTA). 

### 2.2. FACS Analysis of Annexin V Binding and Forward Scatter

After incubation under the respective experimental condition, 50 µL cell suspension was washed in Ringer solution containing 5 mM CaCl_2_ and then stained with Annexin V FITC (1:200 dilution; ImmunoTools, Friesoythe, Germany) in this solution at 37 °C for 20 min under protection from light. Annexin V binding cells were defined according to the gating shown in the respective figure. In the following, the forward scatter (FSC) of the cells was determined, and annexin V fluorescence intensity was measured at an excitation wavelength of 488 nm and an emission wavelength of 530 nm on a FACS Calibur (BD, Heidelberg, Germany).

### 2.3. Measurement of Intracellular Ca^2+^

After incubation, erythrocytes were washed in Ringer solution and then loaded with Fluo 3/AM (Biotium, Hayward, USA) in Ringer solution containing 1 mM CaCl_2_ and 2 µM Fluo 3/AM. The cells were incubated at 37 °C for 30 min and washed twice in Ringer solution containing 1 mM CaCl_2_. The Fluo 3/AM loaded erythrocytes were resuspended in 200 µL Ringer. Then, Ca^2+^ dependent fluorescence intensity was measured at an excitation wavelength of 488 nm and an emission wavelength of 530 nm in FACS analysis.

### 2.4. Measurement of Hemolysis

For the determination of hemolysis, the samples were centrifuged (3 min at 400 g, room temperature) after incubation, and the supernatants were harvested. As a measure of hemolysis, the hemoglobin (Hb) concentration of the supernatant was determined photometrically at 405 nm. The absorption of the supernatant of erythrocytes lysed in distilled water was defined as 100% hemolysis.

### 2.5. Statistics

**Figure 1:**
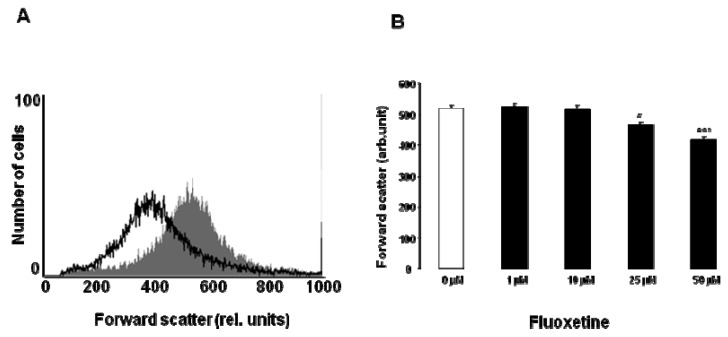
Effect of fluoxetine on erythrocyte forward scatter. (**A**) Original histogram of forward scatter in erythrocytes following exposure for 48 h to Ringer solution without (grey shadow) and with (black line) presence of 50 µM fluoxetine; (**B**) Arithmetic means ± SEM (*n* = 12) of the normalized erythrocyte forward scatter (FSC) following incubation for 48 h to Ringer solution without (white bar) or with (black bars) fluoxetine (1–50 µM). * (*p* < 0.05) and *** (*p* < 0.001) indicate significant difference from the absence of fluoxetine (ANOVA).

**Figure 2:**
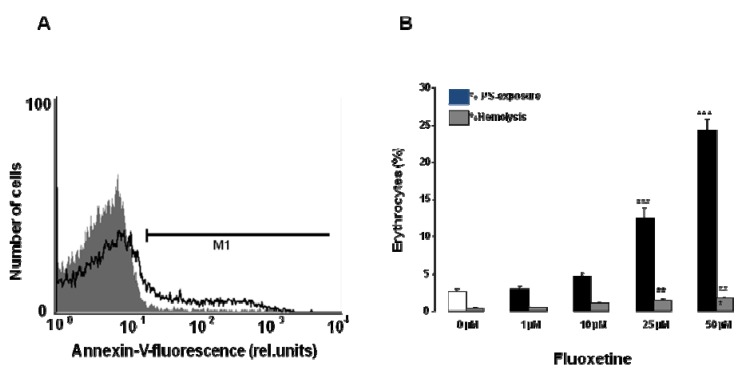
Effect of fluoxetine on phosphatidylserine exposure and hemolysis. (**A**) Original histogram of annexin V binding of erythrocytes following exposure for 48 h to Ringer solution without (grey shadow) and with (black line) presence of 50 µM fluoxetine; (**B**) Arithmetic means ± SEM (*n* = 12) of erythrocyte annexin V binding following incubation for 48 h to Ringer solution without (white bar) or with (black bars) presence of fluoxetine (1–50 µM). For comparison, arithmetic means ± SEM (n = 4) of the percentage of hemolysis is shown as grey bars. ** (*p* < 0.01) and *** (*p* < 0.001) indicate significant differences from the absence of fluoxetine (ANOVA).

**Figure 3:**
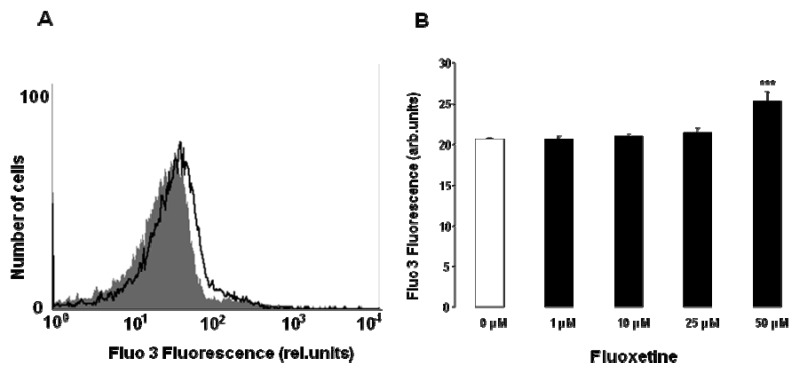
Effect of fluoxetine on erythrocyte cytosolic Ca^2+^ concentration. (**A**) Original histogram of Fluo-3 fluorescence intensity (arbitrary units) in erythrocytes following exposure for 48 h to Ringer solution without (grey shadow) and with (black line) presence of 50 µM fluoxetine; (**B**) Arithmetic means ± SEM (*n* = 12) of the Fluo-3 mean fluorescence intensity (MFI) (arbitrary units) in erythrocytes exposed for 48 h to Ringer solution without (white bar) or with (black bars) fluoxetine (1–50 µM). *** (*P* < 0.001) indicate significant difference from the absence of fluoxetine (ANOVA).

**Figure 4:**
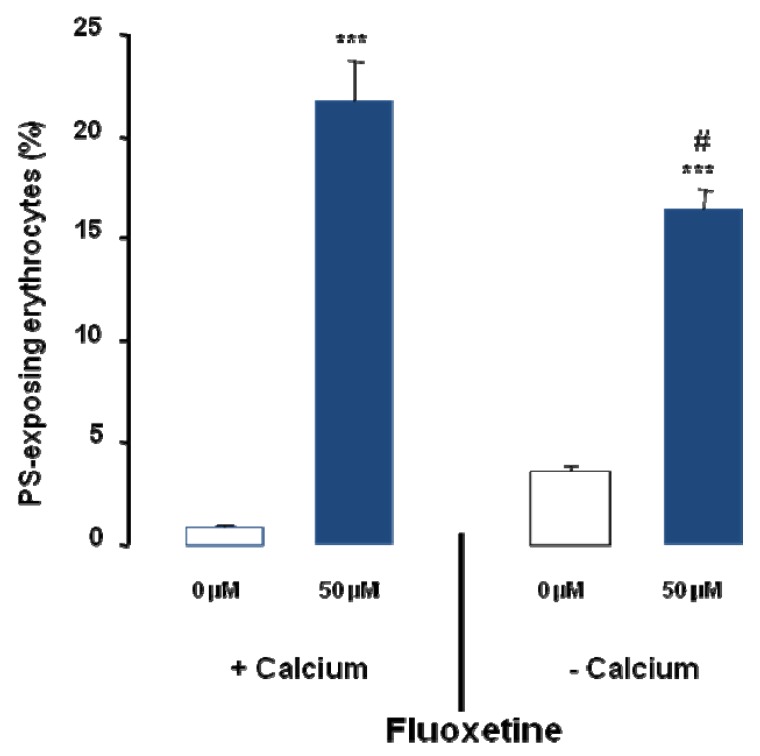
Effect of Ca^2+^ withdrawal on fluoxetine induced annexin V binding. Arithmetic means ± SEM (*n* = 4) of the percentage of annexin V binding erythrocytes after a 48 h treatment with Ringer solution without (white bar) or with (black bars) 50 µM fluoxetine in the presence (left bars, + Calcium) and absence (right bars, − Calcium) of calcium. *** (*p* < 0.001) indicate significant difference from the absence of fluoxetine (ANOVA), # (*p* < 0.05) indicates significant difference from the respective values in the presence of Ca^2+^.

Data are expressed as arithmetic means ± SEM. Fluorescence intensities were shown in logarithmic scale. That means that neither of the populations shown in the histograms of Figure 1, Figure 2, Figure 3, Figure 4 are normally distributed. In Figure 1, Figure 2, cells with elevated fluorescence intensity (annexin-positive of PS-exposing cells) were gated as indicated by the marker. The percentage of PS-exposing cells was calculated for all blood samples. The percentages of PS-exposing cells of the individual blood samples was distributed normally (passed normality test), however, we have used now a non-parametric ANOVA (Kruskal-Wallis Test with Dunn post test using InStat software, Graphpad Software Inc., La Jolla, CA, USA) because there were significant differences among the standard deviations between groups (the scattering increased with increase in fluorescence intensities). n denotes the number of different erythrocyte specimens studied. Since different erythrocyte specimens used in distinct experiments are differently susceptible to triggers of eryptosis, the same erythrocyte specimens have been used for control and experimental conditions.

## 3. Results

The present study analyzed the impact of fluoxetine on eryptosis, and the suicidal death of erythrocytes. A hallmark of eryptosis is cell shrinkage To possibly detect an effect of fluoxetine on cell volume, forward scatter was determined in FACS analysis as a measure of cell volume. As illustrated in Figure 1, a 48 h exposure of erythrocytes to fluoxetine was followed by a slight decrease of forward scatter, an effect reaching statistical significance at 25 µM fluoxetine concentration. As a result, fluoxetine treatment was followed by erythrocyte shrinkage. 

A further hallmark of eryptosis is cell membrane scrambling with breakdown of cell membrane phosphatidylserine asymmetry and exposure of phosphatidylserine at the cell surface. Phosphatidylserine abundance at the cell surface was thus estimated utilizing annexin V binding in FACS analysis. As illustrated in Figure 2, a 48 h treatment with fluoxetine (1–50 µM) increased the percentage of annexin V binding erythrocytes, an effect reaching statistical significance at 25 µM fluoxetine concentration. Thus, fluoxetine triggered cell membrane scrambling.

Further experiments explored whether fluoxetine triggered hemolysis, which was estimated by determination of hemoglobin in the supernatant. As shown in Figure 2, the percentage of hemolysed erythrocytes tended to increase slightly following exposure of erythrocytes for 48 h to fluoxetine, an effect reaching statistical significance at 25 µM fluoxetine concentration (Figure 2). The percentage of hemolysed erythrocytes remained, however, one magnitude lower than the percentage of erythrocytes exposing phosphatidylserine.

Cell membrane scrambling could be triggered by increase of cytosolic Ca^2+^ activity ([Ca^2+^]_i_). Thus, Fluo-3 fluorescence intensity was utilized to estimate [Ca^2+^]_i_. To this end, the erythrocytes were bathed for 48 h in Ringer solution without or with fluoxetine (1–50 µM), loaded with Fluo-3-AM and Fluo-3 fluorescence intensity (arbitrary units) quantified by FACS analysis. As illustrated in Figure 3, a 48 h exposure of human erythrocytes to fluoxetine was followed by an increase of Fluo-3 fluorescence intensity, an effect reaching statistical significance at 50 µM fluoxetine concentration. Thus, fluoxetine treatment was followed by increase of [Ca^2+^]_i_ in human erythrocytes.

In order to define the role of Ca^2+^ in the stimulation of cell membrane scrambling following fluoxetine treatment, experiments were performed either in the presence of 1 mM extracellular Ca^2+^ or in the absence of extracellular Ca^2+^ and presence of the Ca^2+^ chelator EGTA (1 mM). As illustrated in Figure 4, exposure of erythrocytes to 50 µM fluoxetine for 48 h significantly increased annexin V binding, an effect significantly less pronounced in the absence of extracellular Ca^2+^. Accordingly, Ca^2+^ removal significantly blunted the effect of fluoxetine (50 µM) on annexin V binding. However, even in the absence of extracellular Ca^2+^ the percentage of annexin V binding erythrocytes was significantly increased by fluoxetine treatment (Figure 4). Thus, fluoxetine induced cell membrane scrambling was in part but not fully dependent on the presence of extracellular Ca^2+^. 

## 4. Discussion

The present study reveals a stimulatory effect of fluoxetine on eryptosis, the suicidal death of erythrocytes. The concentrations required to stimulate eryptosis are only moderately higher than the concentrations encountered *in vivo* [32,33]. In patients plasma concentrations of 2–3 µM have been determined [34]. Thus, a stimulation of eryptosis is only expected at toxic dosages of fluoxetine or at a particular sensitivity of the erythrocytes. Sensitivity of individual patients to triggers of eryptosis could be enhanced by disease, intoxication and or treatment with other proeryptotic drugs. Along those lines, eryptosis is triggered by a wide variety of xenobiotics [31,35,36,37,38,39,40,41,42,43,44,45,46,47,48,49,50,51,52,53,54,55,56,57,58,59,60,61,62,63,64,65,66,67] and enhanced eryptosis contributes to the pathophysiology of several clinical disorders [12], such as diabetes [23,68,69], renal insufficiency [70], hemolytic uremic syndrome [71], sepsis [72], malaria [73,74,75,76,77], sickle cell disease [78], Wilson’s disease [76], iron deficiency [79], malignancy [80], phosphate depletion [81], and metabolic syndrome [61].

The fluoxetine induced cell membrane scrambling with breakdown of phosphatidylserine asymmetry of the erythrocyte cell membrane was significantly blunted in the absence of extracellular Ca^2+^ and was thus at least in part due to entry of extracellular Ca^2+^. An increase of cytosolic Ca^2+^ activity ([Ca^2+^]_i_) is the main stimulator of cell membrane scrambling and subsequent phosphatidylserine translocation from the inner leaflet of the cell membrane to the outer leaflet of the cell membrane [12]. The Ca^2+^ entry is accomplished by Ca^2+^ permeable non selective cation channels involving the transient receptor potential channel TRPC6 [13]. The Ca^2+^ permeable cation channels in the erythrocyte cell membrane are activated by oxidative stress [82]. 

The erythrocyte shrinkage following fluoxetine treatment again presumably results from increase of [Ca^2+^]_i_, which activates Ca^2+^ sensitive K^+^ channels [15,83]. The subsequent cell membrane hyperpolarization increases the electrical driving force for Cl^−^ exit and the cellular loss of KCl with osmotically obliged water eventually leads to the observed cell shrinkage [16].

According to Fluo-3 fluorescence intensity, fluoxetine does increase cytosolic Ca^2+^ activity, an effect, however, reaching statistical significance only at high fluoxetine concentrations. The possibility must be considered that the sensitivity of Fluo-3 fluorescence for the measurement of cytosolic Ca^2+^ activity may be limited and thus higher concentrations of cytosolic Ca^2+^ activity are required to significantly detect alterations of Fluo-3 fluorescence intensity than the cytosolic Ca^2+^ concentrations required to trigger cell membrane scrambling resulting in statistically significant increases of annexin V binding. Nevertheless, even in the nominal absence of extracellular Ca^2+^ fluoxetine still significantly increases annexin V binding, *i.e.*, cell membrane scrambling. Thus, additional mechanisms are presumably involved in the stimulation of erythrocyte membrane scrambling by fluoxetine. Eryptosis could be triggered by oxidative stress or weakened antioxidant defence [47,82,84]. Moreover, eryptosis could be triggered by energy depletion [18]. Eryptosis could further be stimulated or fostered by caspase activation [19,20,21,22,23], by stimulation of Janus activated kinase JAK3 [25], casein kinase [26,27], p38 kinase [28] and PAK2 kinases [29] or by inhibition of AMP activated kinase AMPK [14] and cGMP dependent protein kinase [24]. 

A stimulation of eryptosis may result in anemia. Eryptotic erythrocytes are rapidly removed from circulating blood [12]. As long as the accelerated loss of erythrocytes by stimulated eryptosis is matched by a similar stimulation of erythropoiesis, the blood count may remain almost constant. As soon as the enhanced erythropoiesis cannot compensate for the excessive eryptosis, anemia develops [12]. 

Excessive eryptosis may further lead to adhesion of phosphatidylserine exposing erythrocytes to endothelial CXCL16/SR PSO [85]. At least in theory, erythrocyte adhesion to the vascular wall could compromize microcirculation and thus interfere with blood flow [85,86,87,88,89,90]. The interference of eryptosis with microcirculation may be compounded by the stimulating effect of phosphatidylserine exposure on blood clotting with subsequent triggering of thrombosis [86,91,92]. 

## 5. Conclusions

Toxic concentrations of the antidepressant fluoxetine stimulates eryptosis, the suicidal erythrocyte death. 
